# High Precision Calibration Algorithm for Binocular Stereo Vision Camera using Deep Reinforcement Learning

**DOI:** 10.1155/2022/6596868

**Published:** 2022-03-31

**Authors:** Jie Ren, Fuyu Guan, Tingting Wang, Baoshan Qian, Chunlin Luo, Guoliang Cai, Ce Kan, Xiaofeng Li

**Affiliations:** ^1^College of Physical Education and Training, Harbin Sport University, Harbin 150008, China; ^2^Party and Government Office, Harbin Sport University, Harbin 150008, China; ^3^Winter Olympic College, Harbin Sport University, Harbin 150008, China; ^4^College of Sports Human Science, Harbin Sport University, Harbin 150008, China; ^5^Department of Information Engineering, Heilongjiang International University, Harbin 150025, China

## Abstract

Camera calibration is the most important aspect of computer vision research. To address the issue of insufficient precision, therefore, a high precision calibration algorithm for binocular stereo vision camera using deep reinforcement learning is proposed. Firstly, a binocular stereo camera model is established. Camera calibration is mainly divided into internal and external parameter calibration. Secondly, the internal parameter calibration is completed by solving the antihidden point of the camera light center and the camera distortion value of the camera plane. The deep learning fitting value function is used based on the internal parameters. The target network is established to adjust the parameters of the value function, and the convergence of the value function is calculated to optimize reinforcement learning. The deep reinforcement learning fitting structure is built, the camera data is entered, and the external parameter calibration is finished by continuous updating and convergence. Finally, the high precision calibration of the binocular stereo vision camera is completed. The results show that the calibration error of the proposed algorithm under different sizes of checkerboard calibration board test is only 0.36% and 0.35%, respectively, the calibration accuracy is high, the value function converges quickly, and the parameter calculation accuracy is high, the overall time consumption of the proposed algorithm is short, and the calibration results have strong stability.

## 1. Introduction

At the moment, computer vision is a hot research field. It is widely used in various fields and is particularly useful in UAV visual positioning, robot navigation, and other areas [1, 2]. Binocular stereo vision is based on the premise of mimicking human vision, and it employs two cameras to complete visual measurement using parallax calculations. It offers numerous advantages, including noncontact, high precision, and great concealment. It is capable of meeting people's growing measuring and detecting requirements. Therefore, binocular stereo vision has a promising application future [3]. High precision camera calibration is one of the keys to ensuring the effective functioning of a binocular stereo vision system. As a result, it is vital to investigate the high precision calibration of binocular stereo vision cameras.

The primary goal of binocular stereo vision calibration is to calculate the internal parameters and spatial position parameters of the camera, as well as to determine the correlation between two-dimensional coordinates and three-dimensional coordinates [4], thereby ensuring the accuracy of the vision system measurement. Traditional camera calibration and self-calibration are the two main types of extant camera calibration technologies. The traditional camera calibration method computes the camera's internal characteristics based on a predetermined model and appearance data such as target size. This calibration method has the drawbacks of being difficult to use and being extremely dependent on the equipment. This calibration method has the disadvantages of complex operation and high equipment dependence. The self-calibration of cameras does not need external help, but it only calculates the camera parameters through the feature point data between the target images. Despite the fact that this method is easier than traditional camera calibration, the calibration accuracy is low [5, 6]. Therefore, this paper proposes a high precision calibration algorithm for binocular stereo vision camera using deep reinforcement learning and attempts to combine deep learning and reinforcement learning to give a new concept for binocular stereo vision camera calibration. The main contributions of this paper are as follows: (1) the camera distortion is considered when calculating the internal parameters of the camera to improve the calculation accuracy of the internal parameters; (2) the external parameters of the camera are calculated using deep reinforcement learning algorithm, which fully utilizes the advantages of deep learning and reinforcement learning; (3) the proposed algorithm can effectively complete the high precision camera calibration and has a specific application.

## 2. Related Work

In the field of computer science, binocular stereo vision is a hot topic. The method of camera calibration has been proposed by a number of academics both at home and abroad. Literature [7] proposed an alternative adjustment-based camera calibration algorithm for binocular stereo vision systems, established a binocular vision calibration system with left and right camera coordinates as reference coordinates, and optimized the internal parameters of the two cameras through alternating adjustment experiments to achieve the best value. The optimal distortion parameters and internal and external parameters are then obtained by optimizing all internal and external parameters although the algorithm's convergence time is slow. The deep learning is updated using the projection vector of feature points, and the best translation vector is found using the projection vector of feature points. Literature [8] used the singular value decomposition approach to calculate the relative attitude matrix during the absolute azimuth interpretation stage. The posture estimation problem of a stereo vision measuring system based on feature points is solved, and stereo vision is expanded. In the image, just one pose parameter from the two collected images is optimized. The algorithm is designed in such a way that it does not effectively increase camera calibration accuracy. Literature [9] established and calibrated a heterogeneous binocular stereo vision system, which included a high-definition color camera and an infrared thermal camera system and designed an algorithm for accurate positioning and sorting of calibration points on the calibration plate. The camera is then calibrated, as is the binocular stereo vision system. This method has a low mistake rate, but it takes a long time. Literature [10] demonstrated online calibration of dynamic binocular stereo vision's external parameters for rectangular images of undetermined size. The elliptical pose and heading reference system is used in real time to provide an approximate value of the rotation angle, and the rotation angle of each camera is solved iteratively using only a single rectangular centroid according to the homology map between images. To complete the camera calibration, the yaw angle is corrected according to the matching rectangle prime angle. However, the algorithm's accuracy is low. Literature [11] examined the methods for calibrating the ultra-wide field of view long wave infrared camera's internal and external parameters. In order to address the issues of camera imaging distortion and low resolution, an external parameter calibration method based on the least square method is proposed, and the calibration results of a long wave infrared camera are evaluated in conjunction with the relevant data of internal parameters. Experiments validate the approach's objective correctness. However, its stability is low. Literature [12] investigated the parallel binocular stereo vision system and zoom calibration method. The image information is gathered using the triangulation concept, the baseline accuracy is ensured by moving the camera, the calibration results are produced, and the BP neural network is used to process the calibration data further to increase the visual measurement accuracy. However, due to the characteristics and mutual restrictions of left and right images in binocular stereo vision, this strategy is prone to local optimization, and overall stability is not satisfied. To address the disadvantages of traditional methods, this work investigates the high precision calibration for a binocular stereo vision camera using deep reinforcement learning, with an emphasis on addressing the camera's internal and external parameters. Experiments validate the algorithm's performance, and camera calibration may be accomplished quickly.

## 3. Methodology

### 3.1. Binocular Stereo Vision Camera Model

**Figure 1:**
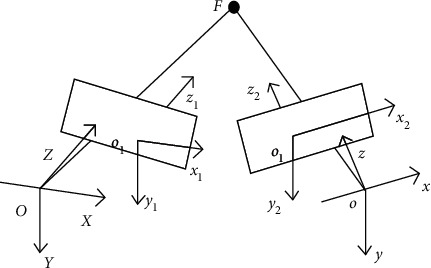
Binocular stereo vision measurement.

Through the imaging lens, the camera translates the projection from three-dimensional coordinates to two-dimensional coordinates. This process is known as imaging transformation, and it is referred to as camera model. The camera model can be used to determine the location relationship between each point on the measured image and the space object [13]. Binocular stereo vision cameras use the parallax principle to obtain image information from left and right cameras.  Figure 1 depicts the positioning and coordinates of the two cameras in binocular stereo vision assessment.  Figure 1 shows the location and coordinates of the two cameras in binocular stereo vision measurement.*O*-*XYZ* represents the coordinate system of the left camera. The origin is located at the start of the global coordinate system. The coordinate system of the left camera image is *o-x*_1_*y*_1_*z*_1_, the coordinate system of the right camera is *o-xyz*, and the coordinate system of the right camera image is *o-x*_2_*y*_2_*z*_2_. The camera transformation model is then developed using the imaging lens principle [14].

(1) $$\begin{array}{c} A_{l}\cdot \left[\begin{array}{c} X\\ Y\\ 1 \end{array}\right]=\left[\begin{array}{ccc} c_{X} & b_{l} & d_{l}\\ 0 & c_{Y} & f_{l}\\ 0 & 0 & 1 \end{array}\right]\left[\begin{array}{c} x_{1}\\ y_{1}\\ z_{1} \end{array}\right],\\ A_{r}\cdot \left[\begin{array}{c} x\\ y\\ 1 \end{array}\right]=\left[\begin{array}{ccc} c_{x} & b_{r} & d_{r}\\ 0 & c_{x} & f_{r}\\ 0 & 0 & 1 \end{array}\right]\left[\begin{array}{c} x_{2}\\ y_{2}\\ z_{2} \end{array}\right], \end{array}$$

where *A*_*l*_ and *A*_*r*_ represent the image scale coefficients of the left and right cameras, and *c*_*X*_ and *c*_*x*_ represent the scale coefficients of the left and right cameras. Using axis *d* and axis *f* as measurement scales, *d*_*l*_, *f*_*l*_, *d*_*r*_ and *f*_*l*_ are the optical center of left and right cameras, while *b*_*l*_ and *b*_*r*_ are the error coefficients in vertical direction of left and right cameras.

There will be some translation and rotation during the pixel location conversion of the left and right cameras. The original position coordinate of the target object is designated as *K*(*x*_*k*_, *y*_*k*_, *z*_*k*_), and a corner of the target object is chosen for translation.

Comparing the corresponding coordinates of the corner point before and after the pose transformation of the target object, the translation matrix *T* can be obtained, and the calculation Equation is as follows:

(2) $$\begin{array}{c} T=\left[\begin{array}{c} x_{k}^{\prime }-x_{k}\\ y_{k}^{\prime }-y_{k}\\ z_{k}^{\prime }-z_{k} \end{array}\right], \end{array}$$

where *K*(*x*_*k*_′, *y*_*k*_′, *z*_*k*_′) is the corner position of the target object after translation.

Assuming that the target object's rotation angles along the global coordinate system *O*-*X*_0_*Y*_0_*Z*_0_ are *γ*, *λ* and *μ*, respectively, the rotation matrix for different angles of rotation around the *X*_0_, *Y*_0_ and *Z*_0_ axes can be expressed as

(3) $$\begin{array}{c} R_{X_{0}}=\left[\begin{array}{ccc} 1 & 0 & 0\\ 0 & \cos\;\gamma & \sin\;\gamma \\ 0 & -\sin\;\gamma & \cos\;\gamma \end{array}\right], \end{array}$$

(4) $$\begin{array}{c} R_{Y_{0}}=\left[\begin{array}{ccc} \cos\;\lambda & 0 & -\sin\;\lambda \\ 0 & 1 & 0\\ \sin\;\lambda & 0 & \cos\;\lambda \end{array}\right], \end{array}$$

(5) $$\begin{array}{c} R_{Z_{0}}=\left[\begin{array}{ccc} \cos\;\mu & \sin\;\mu & 0\\ -\sin\;\mu & \cos\;\mu & 0\\ 0 & 0 & 1 \end{array}\right]. \end{array}$$

If a rotation of an angular value is made around a fixed axis, the rotation matrix can be regarded as a superposition of the rotations of *X*_0_, *Y*_0_ and *Z*_0_ as rotation axes.

Equation (3) can be used to calculate the relationship between the initial pose coordinate *k* of the target object's corner and the transformed pose coordinate *k*′ [15]:

(6) xk′yk′zk′=RxX0kRyX0kRzX0k+T.

### 3.2. High Precision Calibration Algorithm for Binocular Stereo Vision Camera

Camera calibration is the process of comparing the camera system to the measurement standard and determining the camera parameters through coordinate and related factor calculations [16, 17]. From two-dimensional data, camera calibration can determine the true location state of the measured object. It is not only a significant step in computer vision research, but it is also a necessary connection in binocular vision noncontact measurement. The accuracy of the stereo vision measurement method is directly affected by whether the computation is accurate or not [18, 19].

Internal parameter calibration and external parameter calibration are the two primary types of camera calibration.  Table 1 describes the parameters.

External parameters are used to determine the position relationship of camera coordinate system, including rotation matrix and translation matrix. The degrees of freedom of translation matrix and rotation matrix are three, respectively, and a total of six camera external parameters are obtained by adding. These external parameters usually need to be obtained by experimental calculation [20]. The parameter calculation process can be regarded as camera calibration. Internal camera parameters, such as focal length, optical center, nonvertical factor, and distortion parameters involved in perspective translation, are included in Table 1. External parameters such as the rotation matrix and translation matrix are used to determine the position connection of the camera coordinate system. The degrees of freedom of the translation matrix and rotation matrix are three, respectively, and adding them yields a total of six camera external parameters. These external parameters are normally derived through experimental calculation [20]. The process of calculating parameters might be thought of as camera calibration.

#### 3.2.1. Internal Parameter Calibration

There will be an intersection point between the parallel line and the infinite plane, which is known as the blanking point, according to projective geometry theory. The existence of the blanking point is determined by the line's direction. According to this theory, a blanking point must exist between the camera's optical center and the camera plane. The blanking points can be used to calibrate the camera's internal parameters. It is assumed that there are two blanking points on the camera plane, *g* and *h*, in the vertical and parallel directions, respectively, which are connected to the camera's optical center *O* to produce *OG* and *OH*. If the coordinate of the camera's principal point is (*d*, *f*) and the coordinates of the blanking points *G* and H are (*g*, *h*), then

(7) $$\begin{array}{c} OG={\left(\left(g_{i}-d\right)c_{X},\left(h_{i}-f\right)c_{x},f_{0}\right)}^{T}, \end{array}$$

(8) $$\begin{array}{c} OH={\left(\left(g_{j}-d\right)c_{X},\left(h_{j}-f\right)c_{x},f_{0}\right)}^{T}, \end{array}$$

where *f*_0_ is the focal length of the camera, and *T* is the transpose symbol.

*G* and *H* are orthogonal fading point pairs, as the following Equation:

(9) $$\begin{array}{c} OG\cdot OH=0. \end{array}$$

Calculation Equation of hidden points cancelled is shown in equation (8).

(10) $$\begin{array}{c} \left(g_{i}-d\right)\left(g_{j}-d\right){\left(c_{X}\right)}^{2}+\left(h_{i}-f\right)\left(h_{j}-f\right){\left(c_{x}\right)}^{2}+{\left(f_{0}\right)}^{2}=0. \end{array}$$

The internal parameter calibration of the camera can be accomplished preliminary using equation (8). The camera model is typically split into linear and nonlinear models based on the imaging geometric connection. However, the premise of the linear model is based on an ideal assumption, which can only simply express the relationship between image coordinates and spatial coordinates [21]. There will be distortion and camera deformity throughout the actual filming process owing to the influence of numerous circumstances. The real imaging position is (*U*_1_, *V*_1_) if the imaging position in the linear model is (*U*, *V*).

(11) $$\begin{array}{c} \left\{\begin{array}{c} U_{1}=U+\beta ,\\ V_{1}=V+\alpha , \end{array}\right) \end{array}$$

where *β* and *α* are distortion value in transverse and longitudinal imaging direction.

Radial and tangential distortion are the most common types of camera distortion. The tangential distortion is usually minor and unnoticeable. As a result, the radial distortion polynomial is used to express the camera distortion value.

(12) $$\begin{array}{c} \left\{\begin{array}{c} \beta =\chi pr^{2}\left(U_{1}-d\right),\\ \alpha =\chi pr^{2}\left(V_{1}-f\right), \end{array}\right) \end{array}$$

where *χ* represents the radial distortion parameter of the camera. *p* represents the tangential distortion parameter, and *r* represents the radial distortion distance dominated by the image center.

#### 3.2.2. External Parameter Calibration using Deep Reinforcement Learning

Internal parameters are used to calibrate the camera's external parameters. In general, the precision calibration board is chosen to compute the corresponding relationship between camera coordinates and spatial coordinates, as well as to define the structural parameters of the binocular vision system. For external parameter calibration, the deep reinforcement learning algorithm is applied in this study.

**Figure 2:**
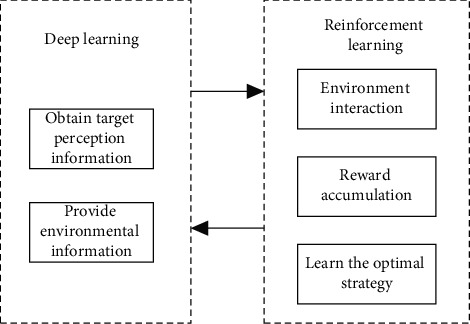
Deep reinforcement learning framework.

The deep reinforcement learning algorithm is a new algorithm that was created by combining deep learning and reinforcement learning. It not only has deep learning's feature extraction ability, but also has reinforcement learning's decision-making power. The traditional reinforcement learning algorithm's applicability space is narrow and discrete. Reinforcement learning effectively overcomes the limitation that it cannot be applied to high-dimensional data analysis by optimizing deep learning, allowing it to be well applied to vast spaces practical scenes [22].  Figure 2 shows the deep reinforcement learning framework.

The goal of reinforcement learning, as shown in Figure 2, is to learn the best approach through environmental interaction and reward accumulation. It is a constant process in which agents interact with their surroundings in order to attain their objectives. The camera external parameter calibration process can be seen as a reinforcement learning problem, and the optimal parameters can be determined as much as feasible through the camera target and coordinate analysis, according to the description of reinforcement learning.

At the moment, classical reinforcement learning can be classified into three types: value-based reinforcement learning, policy-based reinforcement learning, and actor critical learning, which combines value and policy. The actor critical method is a hybrid of the two ways, having the benefits of the policy method for generating actions and dealing with continuous actions, but it requires the calculation of the value function. As a result, in this study, the actor critical method is chosen to calibrate the camera's external settings. The value function must be calculated, and deep learning is a powerful function calculation tool. When applying deep learning to reinforcement learning, however, it is necessary to use a neural network to fit the mapping relationship, which will form a very complex mapping relationship network, and the parameters must be adjusted continuously, implying that the adjustment and convergence of the value function have become a critical problem. As a result, in order to tackle this challenge, this work examines the structure fitting of deep reinforcement learning.

*(1) Deep Reinforcement Learning Structure Fitting*. Deep learning fitting value function, namely, deep reinforcement learning structure fitting, is used in the process of merging deep learning and reinforcement learning in order to fully use the function of reinforcement learning. The study of the deep reinforcement learning structure fitting problem is mostly accomplished by enhancing value function calculation, which is embodied in the adjustment of value function parameters and convergence of value function by building target network.

The estimating procedure of the state action value function is frequently done in practice using function approximation, which is stated as

(13) $$\begin{array}{c} Q\left(q,a,\varpi \right)=Q^{\prime }\left(q,a\right), \end{array}$$

where *Q*(*q*, *a*) is the state action value function, where *q* denotes the state, *a* denotes the action value, and *ϖ* denotes the value function's parameter, which is the reinforcement learning parameter. Equation (13) shows the update method for the value function parameter *ϖ*.

(14) $$\begin{array}{c} \varpi =\varpi_{0}+\varphi \nabla Q\left(q,a\right), \end{array}$$

where *ϖ*_0_ is the initial value of the function parameter, and *φ* is the update coefficient of the value function.

To finish the neural network training, it is required to constantly update the parameters while using a neural network to calculate the value function. This parameter is the value function's parameter. To adjust to the optimal parameters [23, 24], the target network is built, and the parameters are updated in hard and soft modes. When the network unit size must be rigorously controlled, it is considered hard mode. The operating steps are fixed in hard mode. Following the completion of this step, the network parameters are updated by copying. When the network unit size is affected by the overall division unit size, it is considered soft mode, and the update value is minimal in soft mode. The target network parameters (neural network parameters) can then be updated and stated as equation (14).

(15) $$\begin{array}{c} \theta^{\prime }=\left\{\begin{array}{cc} \theta , & \text{if hard},\\ \left(1-\eta \right)\theta +\eta \theta , & \text{if soft}, \end{array}\right) \end{array}$$

where *θ* denotes the neural network's initial parameters The updated neural network parameters are denoted by *θ*′, and the value function is denoted by equation (15).

(16) $$\begin{array}{c} \varpi^{\prime }=\left\{\begin{array}{cc} \varpi , & \text{if hard},\\ \left(1-\eta \right)\varpi +\eta \varpi , & \text{if soft}, \end{array}\right) \end{array}$$

where *ϖ*′ is the updated value function parameters, and *η* is a small value in soft mode, which can help update the parameters properly.

According to the equation (15), after *n* iterations, the value function has the following equation (16).

(17) $$\begin{array}{c} \varpi_{0}⟶\varpi_{1}⟶\cdots ⟶\varpi_{n}. \end{array}$$

The parameter convergence of the value function can finally be accomplished after equation (16), that is,

(18) $$\begin{array}{c} \underset{n⟶\infty }{\lim}\varpi_{n}=\varpi . \end{array}$$

*(2) Camera External Parameter Calibration*. The binocular stereo vision camera data is input, and the external parameters of the camera are calibrated using deep reinforcement learning calculations based on the fitting structure.

  Input: sample data is collected by a binocular camera;
  Output: camera external parameter calibration results.

Reinforcement learning parameters are expressed as value function parameters, and the initial reinforcement learning parameter is *ϖ*, the initial value of neural network parameters is *θ*, the deep reinforcement learning structure and related parameters are initialized, and the deep reinforcement learning binocular stereo camera parameters are calibrated. Deep reinforcement learning is used to calibrate the parameters of a binocular stereo vision camera.

**Figure 3:**
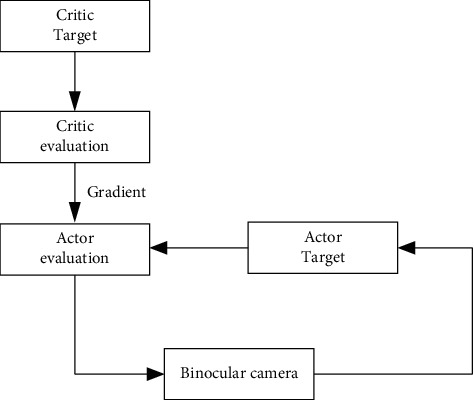
Deep reinforcement learning fitting structure.

1. Half of the binocular stereo vision cameras in the experimental data set were chosen to collect target data as training samples
2. The numbers of hidden layers and nodes of the neural network are determined based on the size of the training samples
3. The fitting structure of deep reinforcement learning is constructed, as shown in Figure 3
4. The neural network is utilized to fit the camera data in order to obtain the value function, and the target network's value function and parameters are established
5. Repeat the iterative value function and neural network, using equations (15) and (16) to adjust the reinforcement learning parameter *ϖ*′ and neural network parameter *θ*′ until the parameters converge
6. Recollect the binocular stereo vision camera data as test data, enter it into the deep reinforcement learning structure, and calculate the camera's external parameters, including the rotation matrix and translation matrix
7. The calibration of the camera's external parameters is complete
8. End

## 4. Experimental Analysis and Results

To evaluate the performance of the binocular stereo vision camera's high-precision calibration algorithm based on deep reinforcement learning, an experimental binocular stereo vision system is constructed.

### 4.1. Experimental Environment

The vs2019 development platform has been completed. The simulation data is run on Windows 10, and the algorithm is developed in opencv2.49.  Table 2 shows the experimental apparatus, which consists of two cameras, two chess and card grid calibration boards, and a computer.

**Figure 4:**
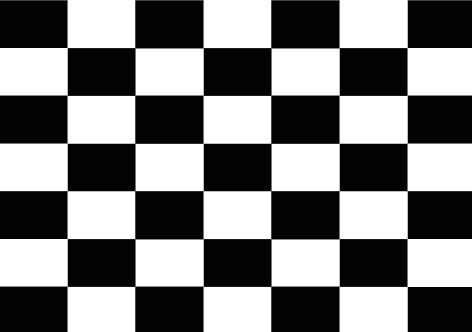
Chess and card grid calibration board.

AutoCAD software is utilized in the experiment to construct chess and card images, develop and print them, and create a calibration board, as illustrated in Figure 4.

### 4.2. Data Set

The experimental data are drawn from two common data sets as well as a visual system measurement data set: the KITTI data set, the cityscapes data set, and the visual system measurement data set. The KITTI data set is the world's largest automatic driving scenario visual measurement dataset, and it is utilized for visual ranging, target detection, and tracking. The data gathering platform is outfitted with four cameras, one sensor, and one GPS navigation system to collect image data in a variety of scenarios such as cities, towns, and roads, including 389 pairs of stereo images and optical flow diagrams. The cityscapes data set is of a vast order of magnitude, containing street stereoscopic images of 50 distinct cities as well as numerous pixel level annotations, including 5,000 high-quality pixel level annotations and 20,000 poor annotations. The data set is ideal for training deep neural networks. A vision system measurement data set: the vision system collects stereoscopic images of six streets using binocular cameras, yielding a total of 20,000 images with a pixel resolution of 1280 × 960. During the experimental test, 1000 images are chosen from each of the three data sets mentioned above, for a total of 3,000 images evaluated. The first half of the data is utilized to train deep reinforcement learning algorithms, while the other half is used for experimental testing.

The studies were performed in the same noise and light environment to ensure the image acquisition impact. Two groups of studies were conducted, each with a 10 mm and 20 mm chess and card grid calibration board. The binocular stereo vision system captured a total of 1,000 images. At the same time, the collected image is filtered and preprocessed to strengthen the image edge information in order to increase image quality and prevent interference from external variables such as noise and illumination. To improve calibration board accuracy, the dimensions of the two chess and card grid calibration boards are 10 mm and 20 mm, respectively, and the measurement field of view is 7m × 6 m, chess and card grid calibration plates are randomly placed in the camera system's measurement field, and the spacing between the two calibration plates is 4 m.

### 4.3. Evaluation Criteria

(1) Calibration precision: This study proposes a calibration algorithm with great precision. To validate the algorithm's completion impact, a special comparative examination of calibration accuracy is required. The error is a method of expressing the precision of the calibration results. The calculation Equation is shown in equation (18).
  (19) $$\begin{array}{c} e=\sqrt{{\left(x_{w}-x_{w}^{\prime }\right)}^{2}+{\left(y_{w}-y_{w}^{\prime }\right)}^{2}}, \end{array}$$
where *e* is calibration error. (*x*_*w*_, *y*_*w*_) and (*x*_*w*_′, *y*_*w*_′) represent the real coordinates and measurement coordinates of the target pixel, respectively.
(2) Convergence of value function: Convergence of the value function: the convergence of the value function is one of the keys to realizing the fit between deep learning and reinforcement learning in the use of deep reinforcement learning algorithms. As a result, this experiment draws the value function network loss function curves of several algorithms to ensure that this method is convergent.
(3) Parameter calculation accuracy: Parameter adjustment is also one of the keys to realize the fitting of deep learning and reinforcement learning. Therefore, parameter calculation accuracy is also an effective index to show the performance of the proposed algorithm. The accuracy calculation Equation is as follows:
  (20) $$\begin{array}{c} \text{Accu}=\frac{L_{1}}{L_{\text{tot}}}\times 100\%, \end{array}$$
where *L*_tot_ represents the actual number of parameter calculations. *L*_1_ is the number of correct parameters in the calculation result.
(4) Camera calibration time consumption: Camera calibration is an important prerequisite in the application of binocular stereo vision system. It is very important for the vision system to complete camera calibration quickly.
(5) Stability of calibration results: The stability of the calibration results of the proposed algorithm is compared with those of Literature [7], Literature [8], Literature [9], Literature [11], and Literature [12].The measurement of stability is based on the change of camera calibration result data sequence. It is assumed that the calibration data series has the same keywords. If the relative order of these terms does not change after sorting, the algorithm is stable.

### 4.4. Results and Discussion

#### 4.4.1. Comparison of Calibration Precision

This paper's main goal is to achieve high precision calibration of a binocular stereo vision camera. As a result, the proposed algorithm is compared to the algorithms in Literature [7], Literature [8], Literature [9], Literature [11], and Literature [12] algorithms in order to reflect the efficiency of the algorithm established in this work as shown in Table 3.

It can be seen from Table 3 that the test findings are quite important. The calibration errors of the algorithm are 0.36% and 0.35% for 10 mm and 20 mm chess and card grid calibration plates, respectively. In comparison to other literature, the minimum calibration error of Literature [7] under two chess and card grid calibration boards is 0.90%, the minimum calibration error of Literature [8] under two chess and card grid calibration boards is 1.66%, the minimum calibration error of Literature [9] is 1.94%, the minimum calibration error of Literature [11] is 5.20%, and the minimum calibration error of Literature [12] is 1.74%. When we compare the proposed algorithm with five traditional literature algorithms, we can clearly see the advantages of proposed algorithm, demonstrating that the deep reinforcement learning algorithm used in this paper for camera calibration has very high precision and a better calibration effect than the traditional literature algorithm.

#### 4.4.2. Comparison of Convergence of Value Function

**Figure 5:**
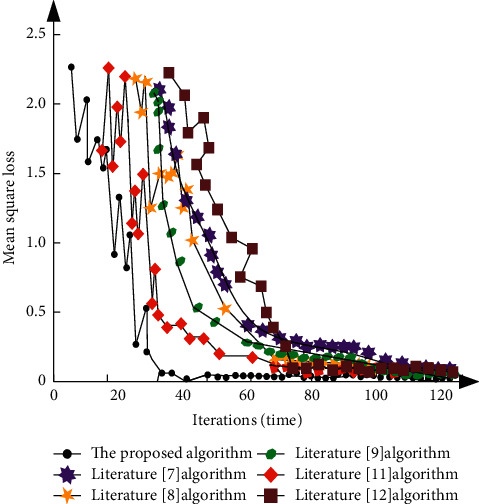
Comparison of loss function curve of value function network.

The loss function curve of the value function network is drawn by using the number of iterations as the abscissa and the mean square loss as the ordinate as shown in Figure 5.

According to Figure 5, each algorithm eventually converges, and the loss of mean square error reduces as the number of iterations grows. When comparing the proposed algorithm's convergence speed to that of the five traditional literature algorithms, it is clear that when the number of iterations is close to 30, the trend of the proposed algorithm's loss function curve begins to gradually tend to be stable, the mean square deviation loss is close to 0, and the value function's convergence is completed. After 70 iterations, the algorithms in Literature [8, 11] and Literature [12] rapidly converge. The convergence of the Literature [7] and Literature [9] algorithms is relatively poor, with a minimum root mean square error of more than 0.2 after convergence. It can be seen that the proposed algorithm's convergence speed is quick, and the convergence effect is good, demonstrating the effectiveness of the value function convergence of the design target network.

#### 4.4.3. Comparison of Parameter Calculation Accuracy

**Figure 6:**
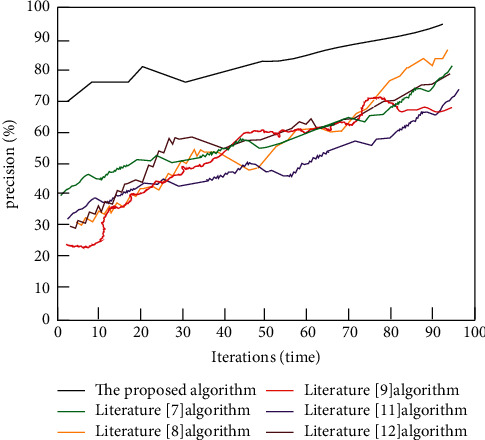
Comparison of parameter calculation accuracy.

This study modifies the value function parameters in reinforcement learning and uses neural network to continually update the parameters to complete the fitting between deep learning and reinforcement learning. The precision of parameter calculation is then critical for camera calibration. It is impossible to acquire accurate calibration results if the accuracy of parameter calculation is low.  Figure 6 depicts the comparison result of parameter calculation accuracy.

The neural network is utilized to update the parameters of the value function, as shown in Figure 6. The modification of the median function of reinforcement learning may be performed with high accuracy through numerous iterations, and the maximum computation high accuracy is about 95%. The algorithm in Literature [9] has a relatively good calculating effect on parameters, with the highest accuracy of around 80%. However, it is still very different from the proposed algorithm. The results of the data comparison can be used to demonstrate the benefits of the proposed algorithm, validate its performance for parameter computation, and ensure the high accuracy calibration of binocular vision camera parameters in this study.

#### 4.4.4. Comparison of Camera Calibration Time Consumption

Table 4 shows the camera calibration time consumption results.

Table 4 shows that when different data sets are used as data sources to assess the calibration time consuming of algorithm, the test results are quite significant. The calibration time consuming of the algorithm in the KITTI data set, cityscapes data set, and vision system measurement data set is 5.2 s, 6.2 s, and 5.3 s, respectively, with an average time consuming of 5.6 s. The proposed algorithm is faster than the average time of algorithms in Literature [7], Literature [8], Literature [9], Literature [11], and Literature [12]. The deep reinforcement learning technique has a very efficient operation rate, which can effectively improve the camera calibration in this work.

#### 4.4.5. Comparison of Stability of Calibration Results

**Figure 7:**
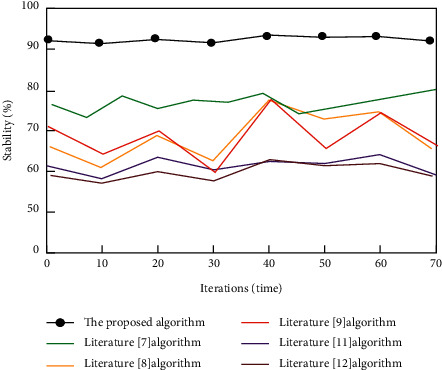
Stability comparison of calibration results.

The stability comparison results of camera calibration results are shown in Figure 7.

According to the data in Figure 7, the proposed algorithm's stability is substantially higher than that of the other five literature algorithms, and the overall stability is controlled at approximately 92%. Among other algorithms, the highest stability of Literature [7], Literature [8], and Literature [9] algorithms is close to 80%, while the stability of Literature [11] and Literature [12] algorithms is almost 60%. This clearly demonstrates the benefits of the proposed binocular vision camera calibration algorithm, which can eliminate external interference and improve algorithm stability.

## 5. Conclusions and Future Works

This paper proposes deep learning to improve reinforcement learning, creates a deep reinforcement learning fitting structure, and investigates the calibration process of a binocular stereo vision camera. The camera's internal and external parameter calibrations are explained in depth, and the proposed algorithm is validated through experimentation. The results show that the proposed algorithm is capable of completing the camera's high precision calibration and has some theoretical utility. This study still has several flaws, and the numerous properties of the camera target are not thoroughly explored. Future works are required to account for target distance, image color, and other parameters, in order to improve the application efficiency and scope of the camera and unlock more possibilities.

## Figures and Tables

**Table 1:** Camera parameter description.

Parameter	Expression	Freedom
Internal parameters	Effective focal length *f*_*u*_, *f*_*v*_.Optical center *u*_0_, *v*_0_	5
	Nonvertical factor *ξ*	
	Radial distortion parameter *χ*_1_, *χ*_2_	4
	Tangential distortion parameter *p*_1_, *p*_2_	

External parameters	Rotation matrix	3
	Translation matrix *T*	3

**Table 2:** Camera performance index.

Performance index	Numerical value
Pixel	1280 × 960
Sampling frequency	60 Hz
Baseline length	60 mm
Focal length	6 mm
Optical dimension	1/3

**Table 3:** Calibration errors of different algorithms.

Algorithms	Chess and card grid calibration board (mm)	
	10	20
The proposed algorithm	0.36	0.35
Literature [7] algorithm	0.90	1.32
Literature [8] algorithm	1.66	3.27
Literature [9] algorithm	1.94	2.58
Literature [11] algorithm	5.65	5.20
Literature [12] algorithm	1.74	4.22

**Table 4:** Comparison of camera calibration time consumption of different algorithms.

Algorithms	Calibration time consuming (s)		
	KITTI data set	Cityscapes data set	Measurement data set of a vision system
The proposed algorithm	5.2	6.2	5.3
Literature [7] algorithm	9.2	16.5	20.2
Literature [8] algorithm	7.1	14.7	21.1
Literature [9] algorithm	8.1	13.0	18.5
Literature [11] algorithm	10.4	11.1	15.4
Literature [12] algorithm	9.1	12.5	49.5

## Data Availability

Readers can access the data supporting the conclusions of the study from KITT data set and cityscapes data set and measurement data set of a vision system.
